# Extrapulmonary tuberculosis in HIV infected patients from the cohort of “Dr Victor Babeş” Hospital, Bucharest, Romania

**DOI:** 10.1186/1471-2334-14-S4-P26

**Published:** 2014-05-29

**Authors:** Simona Tetradov, Sebastian Smâdu, Luminița Ene, Roxana Rădoi, Cristiana Oprea, Ruxandra Burlacu, Dan Duiculescu

**Affiliations:** 1 Clinical Hospital of Infectious and Tropical Diseases “Dr. Victor Babeş”, Bucharest, Romania

## 

The infection with *Mycobacterium tuberculosis* remains the most frequent opportunistic infection in HIV seropositive patients. In Romania the incidence of tuberculosis (TB) in general population is the highest in Europe with 70.9 per 10,000 inhabitants.

We investigated the incidence of extrapulmonary tuberculosis in “Dr Victor Babeş” Hospital cohort and its epidemiological, clinical and outcome particularities.

We performed an observational retrospective study during 2003-2013 among the HIV infected patients from our cohort. We selected the patients with extrapulmonary tuberculosis. The data was obtained from the medical charts and outpatient records.

From 280 cases of confirmed infection we found 55 cases of extrapulmonary tuberculosis. The HIV transmission route was parenteral in 72.55% (95%CI 58.75 to 87.40) of cases. The median age at TB diagnosis was 24 years (95%, CI 20.16 to 25.03) with male/female ratio of 1,21. At the time of TB diagnosis the median CD4 count was 87 cells/cmm (95% CI 72.87 to 131.31). The percent of patients with concomitant pulmonary and extrapulmonary localization was 57.7% (95% CI 40.79 to 72.78). The number of patients with recurrent TB was 17 and 5 had more than one extrapulmonary TB in the studied period. The most frequent extrapulmonary involvement was ganglionar 35/51 (69.7% 95% CI 54.91 to 79.74). The commonest manifestations were fever (57.5% 95% CI 40.79 to 72.78), weight loss (30% 95% CI 17.25 to 47.46) and adenopathy (24.2% 95% CI 12.60 to 41.25) and the median time from the onset to diagnosis was 4 weeks (95% CI 2.611 to 5.209). In 54.5% (95% CI 40.79 to 72.78) of cases the smear was positive, cultures were positive in 69.7% (95% CI 55.61 to 85.10) and in 30% (95% CI 17.25 to 47.46) of cases the diagnosis was made on histopathologic examination. In 45.5% (95% CI 32.50 to 64.78) we obtained an antibiogram that confirmed MDR TB in 11.5% (95% CI 2.37 to 24.34). All patients received treatment and 7.84% (95% CI 6.17 to 31.40) abandoned treatment and 11.76% (95% CI 2.37 to 23.4) died.

Although the extrapulmonary involvement is not very frequent, the diagnosis can be challenging and can take a lot of time especially when it is difficult to obtain a specimen. In a febrile immunodepressed patient extrapulmonary TB should be always considered.

